# The status of bladder cancer research worldwide, a bibliometric review and recommendations

**DOI:** 10.1080/2090598X.2022.2152237

**Published:** 2022-11-30

**Authors:** Hussein Awada, Adel Hajj Ali, Mohammad A. Zeineddine, Hasan Nassereldine, Zahy Abdul Sater, Deborah Mukherji

**Affiliations:** a Faculty of Medicine, American University of Beirut Medical Center, Beirut, Lebanon; b Global Health Institute, American University of Beirut, Beirut, Lebanon; c Division of Hematology and Oncology, American University of Beirut Medical Center, Beirut, Lebanon

**Keywords:** Bladder Cancer, research, bibliometric review, urothelial carcinoma, urology, oncology

## Abstract

**Background:**

Healthcare system costs associated with bladder cancer treatment are among the highest of curable malignancies, and prognosis in advanced disease remains poor. This scoping review examines the worldwide status of bladder cancer research by systematically mapping publications, exploring research topics, support, gaps and limitations that need to be addressed.

**Methods:**

We searched the Web of Science database for publications using controlled vocabulary. Results were limited between 2000–2020, and were included in our study based on pre-specified eligibility criteria. Data used for analysis included author’s names, country of affiliation, language, journal, citations, and funding. Analysis was conducted using Biblioshiny R and SPSS. Research topics were identified according to sub-filters of title words and strings pre-determined by authors.

**Results:**

40,657 results were retrieved, of which 19,976 original articles and reviews met the pre-specified criteria. 92% of the publications originated from 20 countries and were included in the analysis. Trends show an increase across the world, most of which is due to increasing contributions from USA and China. An increase by 1000% in funded publications has been achieved. Studies focused on Surgery, Pathology, and Diagnosis, while Radiotherapy, Palliative care, quality of life and Epidemiology were the least described. Genetics had the most increase while being the most funded. GDP, incidence, prevalence and mortality were each significantly positively correlated with overall bladder cancer research output.

**Conclusion:**

This review described the evolution of bladder cancer research. It also identified significant gaps and limitations that need to be highlighted as priority areas for research investment.

## Introduction

Bladder cancer is the sixth most diagnosed cancer in the United States, representing 3% of all cancers recorded worldwide in 2020 [1,2]. Transitional cell carcinomas, which arise from the urothelial cells lining the inside of the bladder, represent approximately 95% of bladder cancers [3]. Other less common histological subtypes include squamous cell carcinomas (1–2%), adenocarcinomas (1%), small cell carcinomas (<1%) and sarcomas (very rare) [3]. These malignancies typically affect older adults, as 90% of the diagnoses are made in patients over the age of 55, and the average age of diagnosis for this disease is 73 [4].

Tobacco smoking remains the main risk factor for bladder cancer diagnosis, as it increases the risk by two to fourfold when compared to nonsmokers [5]. This relative risk increase following tobacco consumption comes second only to lung cancer [6]. Other risk factors include age, genetics, occupational exposure to aromatic amines and hydrocarbons, ionizing radiation, chlorination and exposure to arsenic in drinking water, as well as drugs such as cyclophosphamide and the antidiabetic agent pioglitazone [7]

The 5-year survival rate for bladder cancer when all stages are included is relatively favorable at 77%, yet merely 5% of patients with metastasis survive beyond the 5-year interval [1]. Furthermore, bladder cancer incidence rates have been on the rise in certain areas of the world, especially Europe [1,6]. These rates are expected to keep increasing in both the developed and developing worlds. While the former’s rising incidence of bladder cancer may be attributed to population growth and aging, the latter’s rise in incidence seems to be secondary to the prevalence of cigarette smoking [6,8]. In addition, the currently burdensome costs inflicted by the disease would be expected to increase with rising rates [9].

Oncology research remains one of the drivers of scientific discovery across the globe, with more than $14 billion being spent annually in its support [10]. The goal in oncology will always be centered around reducing the incidence, morbidity and mortality associated with cancer. Research is the cornerstone upon which further knowledge can be achieved and utilized in realizing these goals. Uncovering the pathogenesis, identifying risk factors, discovering new targets for therapeutic agents and understanding the epidemiological patterns all together serve the implementation of public policies that aim to improve cancer outcomes. Thus, it is essential to positively reinforce these public policies by determining the quantitative and qualitative characteristics of cancer research, as well as how certain research domains evolve and their impact on cancer outcomes.

This scoping review aims to present the results of a bibliometric analysis on the global research on bladder cancer between 2000 and 2020 in the leading countries in cancer research. To our knowledge, this is yet to be looked at and described in the literature. We will try to identify the gaps in bladder cancer research while also assessing the growth in output, international collaboration and funding received for articles investigating bladder cancers. We will also examine the topics addressed by these studies while mapping our results across the developed and developing worlds.

## Methods

We performed a worldwide bibliometric review of the research output related to bladder cancer during the years 2000 to 2020 in the Web of Science (WoS) core collection database, based on articles and reviews. The search was conducted in November 2021. WoS, with its rich dataset, is currently considered as one of the top analytic information and scientific citation platforms, making it the ideal biomedical database for such study.

A comprehensive search was conducted with no language restriction. Publications were selected based on: (a) original articles and reviews (hence excluding abstracts, case series, case reports, letters to the editor, guidelines, commentaries, and narrative reviews), (b) studies tackling the topic of bladder cancer and (c) published in the period of 2000–2020.

A full bibliographic information was collected from WoS for the published articles. Data used for the analysis included authors’ names along with their hospital and country of affiliation, language, journal, number of citations, funding status, and origin. Data analysis was then conducted on Biblioshiny (based on R version 3.6.1, Bibliometrix package version 2.2.1) and Statistical Package for Social Sciences (SPSS) software tools. Categorical variables were analyzed using Chi square analysis and reported as frequency and percentage. The cutt-off for significance was set as 0.05. Data is presented as bar graphs, heat maps, linear regressions and tables.

Further analysis was completed for the top 20 countries. First, the publications of bladder cancer were categorized by their research topics via the use of subfilters based on title words and keywords that were pre-determined by the authors. In cases of doubt, abstracts were reviewed and the research topics were determined based on the study objectives. The research topics used for analysis were diagnosis, chemotherapy, radiotherapy, genetics, epidemiology, screening, palliative care and quality of life, surgery, biomarkers, immunotherapy and targeted therapies, pathology and diagnosis. The categories were not mutually exclusive, thus articles comparing surgery to chemotherapy for example would be included in both categories.

In addition, we compared the number of publications per country relative to the following factors: (a) Gross domestic product (GDP), (b) population size, (c) bladder cancer incidence, (d) mortality and (e) prevalence during the last 5 years.

## Results

### Characteristics and mapping of the articles

**Figure 1.**
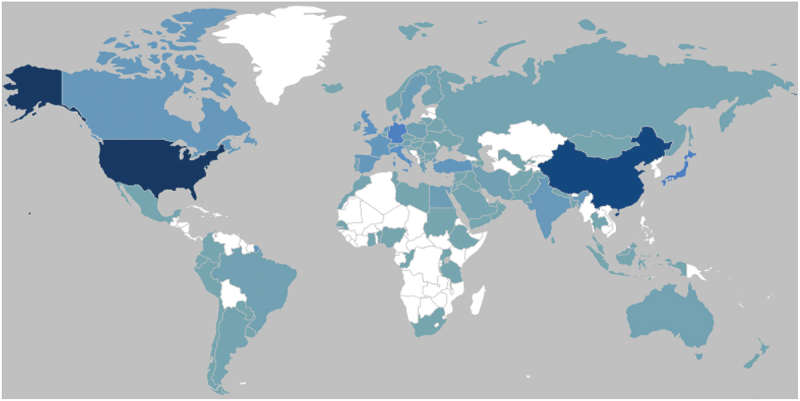
Geographical representation of the global research output on bladder cancer (by corresponding authors).

Our search yielded a total of 19,976 articles worldwide in the period of 2000 to 2020. The publications originated from a total of 103 countries (Figure 1). Most of the publications, however, were concentrated in sparse list of countries, with 92% of the total publications originating from the following 20 countries in descending order: USA, China, Japan, Germany, Italy, United Kingdom, South Korea, Turkey, France, Spain, Canada, India, Netherlands, Sweden, Egypt, Greece, Denmark, Iran, Switzerland, and Austria. In those 20 countries, most of the publications were single country publications with no collaborations from outside.

**Figure 2.**
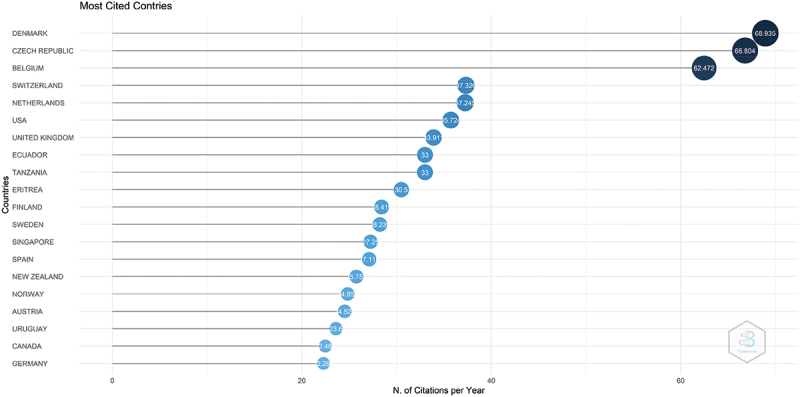
Top countries with the highest average citations per year.

The countries with the highest average of citations per year are listed in (Figure 2), with Denmark, Czech Republic and Belgium being the top 3.

When addressing the authors by the institution of origin, Memorial Sloan Kettering cancer center was the most active institution in bladder cancer publications, followed by China Medical University, Sun Yat-sen University, The University of Texas MD Anderson Cancer Center, and The University of Texas.

The articles were published in a variety of journals worldwide, with Journal of urology (1021) as the leading journal followed by Urology (735), BJU international (702), urologic oncology seminars and original investigations (631), and European urology (462).

The majority of the articles were published in English (19,178), followed by German (300), French (232) and Spanish (144).

**Figure 3.**
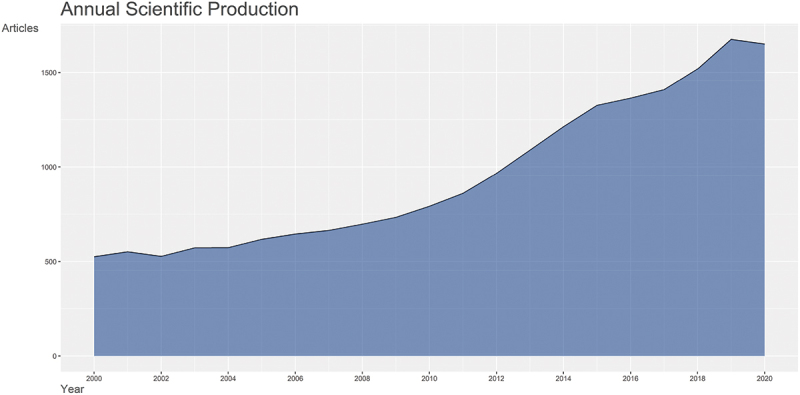
2000–2020 bladder cancer research output per year.

**Figure 4.**
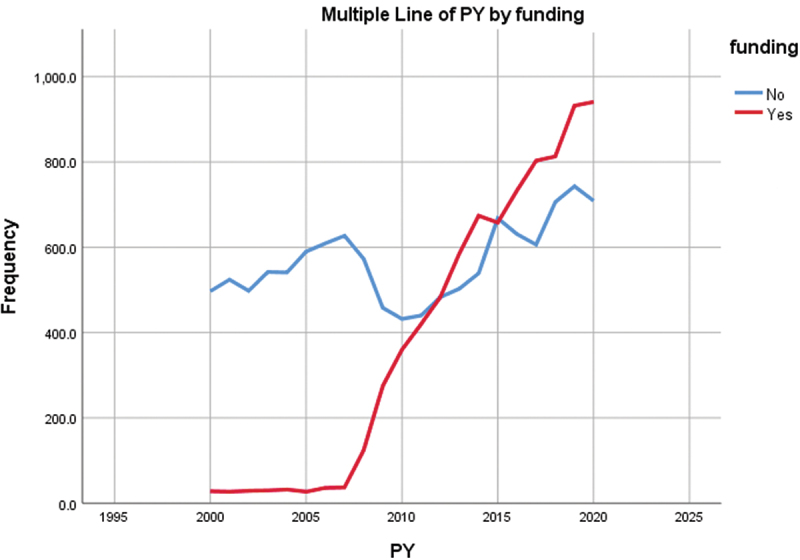
Evolution of funded and non-funded bladder cancer articles by year.

Figure 3 demonstrates the evolution of the bladder cancer publications output from 2000 to 2020, which shows a steady increase in the output across the years. The 2 countries with the largest slope in increment are China and the USA. Figure 4 further highlights the evolution of research output while separating the articles based on the funding status. A remarkable rise in the funded articles was noted in 2007 to equalize the number of non-funded articles in 2012 and then surpass it for the rest of the decade except for the year 2015.

### Research topics

**Figure 5.**
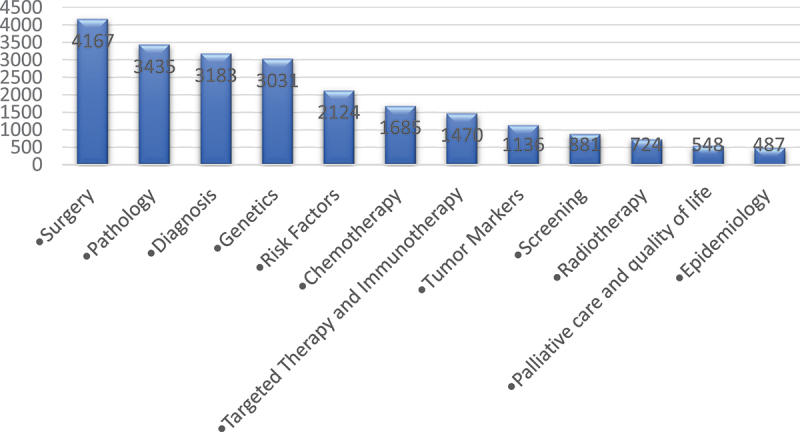
Distribution of the articles across different research topics.

**Figure 6.**
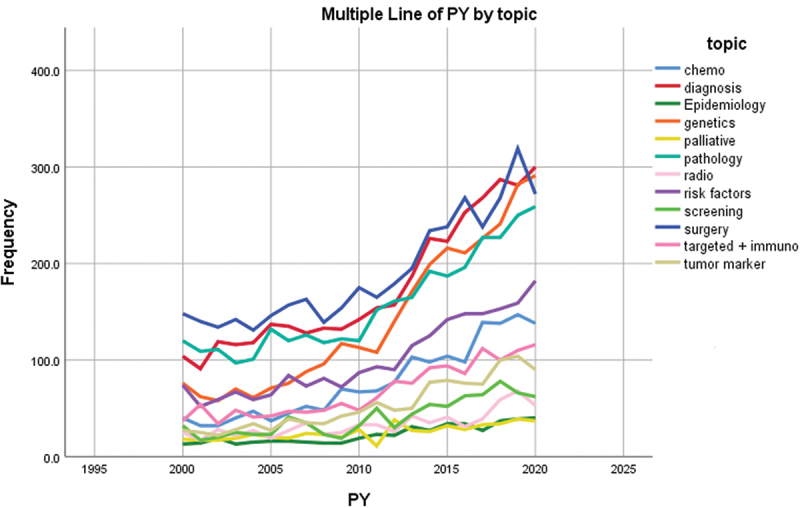
Evolution of the research topics across the years 2000–2020.

A variety of research topics concerning bladder cancer were tackled by the articles (Figure 5); with Surgery, Pathology and Diagnosis making a major chunk of the total publications while Radiotherapy, Palliative care and quality of life and Epidemiology being the least described topics. Furthermore, when addressing the evolution of the research topics across the years, an increase is noted in general for all topics; however, it’s mostly noteworthy for papers addressing Genetics, while barely notable for Epidemiology and Palliative care and quality of life (Figure 6).

**Figure 7.**
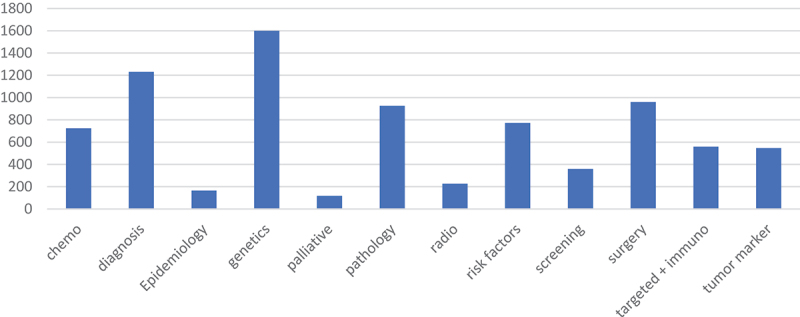
Number of funded articles across the different research topics.

Moreover, a differential distribution of the funding was noted among the different research topics (Figure 7), with genetics taking the biggest part of the funds (19.5%), followed by diagnosis (15%) and pathology (11.3%) while the least funded were Radiotherapy (2.8%), Epidemiology (2%), and Palliative care and quality of life (1.4%).

### Epidemiology

**Figure 8.**
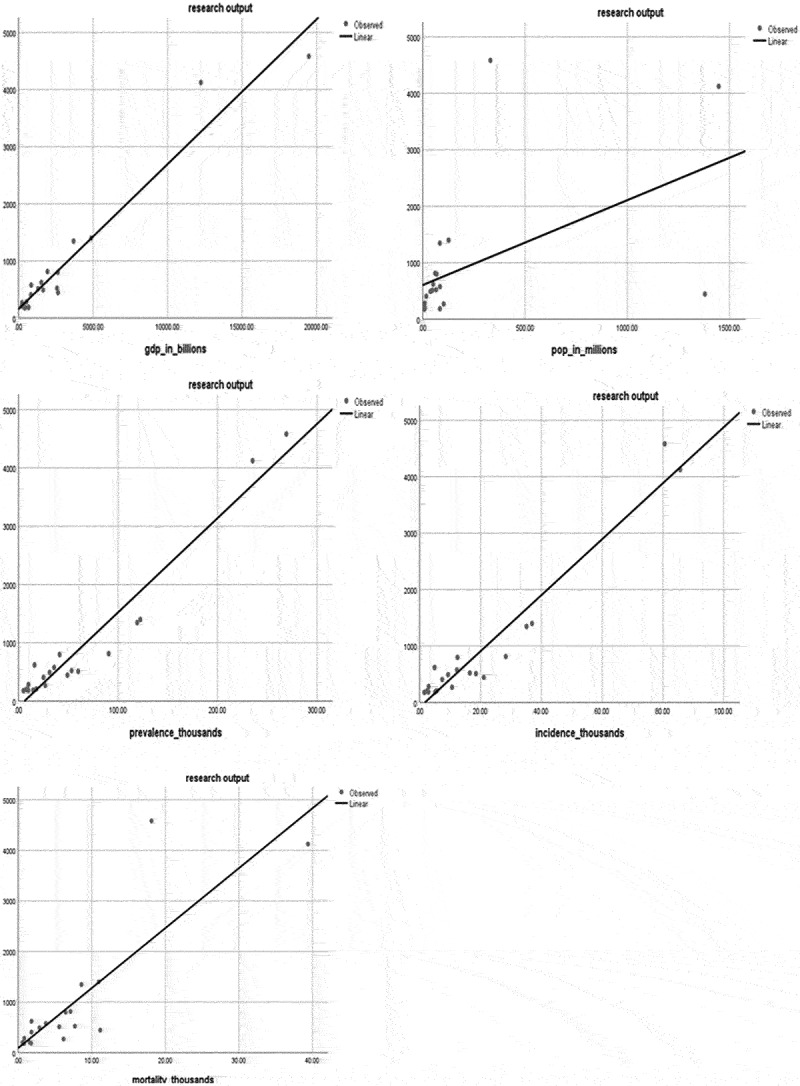
Linear regressions of bladder cancer research output vs GDP, populations size, bladder cancer incidence, prevalence and mortality the last 5 years.

The bladder cancer research output in the top 20 countries, representing 92% of the total publications, was assessed against several epidemiological characteristics of the 20 countries; these were GDP, population size, bladder cancer incidence, prevalence and mortality in the last 5 years. Graphs of the 5 linear regressions are presented in (Figure 8) and they show a strong, significantly positive correlation between the research output and the 5 epidemiological factors (p < 0.0001) for all factors, except for population size (p = 0.021).

## Discussion

The analysis of our results uncovers both gaps and aiding factors while also providing a comprehensive review of the status of bladder cancer research in the top 20 countries that contribute to this domain. Our search of Web of Science showed that these countries are responsible for 92% of the research output. Though we hoped for a more diverse and inclusive contribution across the globe, yet this finding does not come as a surprise, as the same countries have been shown to be amongst the top 25 leading countries in oncology research overall [11].

On a positive note, the output of research studies investigating this topic has more than tripled over the last two decades, and this conforms with the witnessed and ongoing increase in oncology research in general [11]. This growth in research volume is likely related to the ongoing increase in support as reflected by the sharp increase in funding for bladder cancer studies. Our data shows that financially supported articles have increased by more than tenfold, from 5.3% to 57%, as compared to non-supported articles between 2000 and 2020. Funding has long been established as an important factor that influences the quantity, impact, and citations of research articles [9,12]. Per our analysis, the increase in funding was statistically significant across all types of research topics that we investigated. But despite the increase in financial support, bladder cancer research remains underfunded relative to its burden when compared with other types of cancers [13,14]. In addition, our analysis shows that a remarkable discrepancy exists when funding is compared across the selected countries. Only China possesses a positive balance of funded to non-funded articles. This means that bladder cancer still lags when it comes to both funding and the relative commitment towards research. Therefore, an even more drastic increase in available funds is required so that bladder cancer research can catch up with the rest of the field of cancer research as well as with the true burden of the disease.

The global relative of commitment towards bladder cancer compared to total oncology research has decreased from 1.75% to 1.39% during this time frame [10]. Moreover, the analysis of individual country output growth shows that only China and USA had a remarkable increase in bladder cancer research, with the former overcoming the latter as the leading country in bladder cancer research and in concordance with the rapid growth in China’s cancer research output [15]. The growth in bladder cancer research in China is likely a response to its notably increasing health burden associated with bladder cancer [15,16]. The rising incidence and mortality secondary to the rapid demographic transition that China is experiencing, as well as the increasing prevalence of tobacco consumption and air pollution [17,18].

On the other hand, research quality should be considered just as important as the volume of research produced. Hence, we examined the evolution of the quality and scientific impact of articles in this field by using the average citation per paper per year between 2000–2020 as a predictive parameter [19]. The average citations per year parameter has also been shown to reflect the number of authors in the field [19]. Our results show that there has been an overall increase in citations over the last two decades, with the last few years recording 1.5–2 times the average citations per paper of the year 2000. It also showed that Denmark, Netherlands and Switzerland to be having the highest mean of citations per paper. In contrast, China is in the lower quartile in average citations while being on top in article output. Nevertheless, average citations are also impacted by years since articles’ publication, and this may have underestimated the true value of the recent growth spurt in research contributed by China. In addition, the relatively low international collaboration in research between China and the rest of the world may have also affected citations [20].

Still, calls for improvements in research quality and value should always be raised as we aim to advance our understanding and management of bladder tumors. Sharing resources to support transparency and openness can increase the credibility and reliability when the methodology or context are in doubt [21,22]. Other considerations should include how significant results may be better advertised and integrated into clinical practice in order to avoid their shadowing and potential duplication in research work by other investigators [23,24]. Establishing and strengthening the existing international collaborations can also help in improving the quality by expanding the possibilities for innovation and diversifying research expertise, experiences and resources [25].

One of the most important parameters to investigate when evaluating cancer research is the distribution of output in its various topics. It allows us to understand the current observed trends in research and shed the light on the neglected parts that if studied may improve the outcomes for cancer patients. The main area of focus as determined by our results was management, especially surgical interventions. This is mainly because early clinical trials proved that radical cystectomy is superior to bladder sparing treatments and with more focused research ever since solidified surgery as the mainstay of treatment [26]. Second to surgery, more evidence through the growing research on chemotherapy has shown it to be an effective treatment but only when coupled with surgery as either a neoadjuvant or adjuvant treatment [27]. Radiotherapy, which is the least researched part of the management, is most of the time reserved as palliative treatment for terminal cases that refuse surgery [26]. Another heavily researched topic is bladder cancer diagnosis. Advancements in diagnostics of bladder cancer have decreased the risk of recurrence and has improved the early detection of the disease; however, it is yet to improve the survival rate [28]. Better outcomes are further expected with the continuous research and the emergence of new techniques [28].

In the era of personalized medicine, more emphasis is being placed on the genetics and biomarkers of bladder cancer, as advancement in bladder cancer genomics leads to better classification of the disease pathology. This, in turn, paves the way to pathology-tailored management and more accurate prognostic evaluation [29,30]. Nevertheless, many questions in bladder cancer remain unanswered and warrant further unmasking of genetic and other biomarkers features that allow for such tailored management [30]. On the other hand, screening and palliative care remain overlooked and under-investigated. Its early detection and low mortality rate may decrease the significance of screening and palliative care in the management of the disease [1]. However, this should not negate the importance of palliative care for the patients that present with advanced disease and have poor prognosis.

Finally, we looked at the effect of disease burden (incidence, prevalence, mortality), GDP, and population size on research output. There is a significant increase in publication count as the incidence, prevalence, and mortality due to bladder cancer increase. Such an observation is expected since countries with increasing burden to bladder cancer are likely to allocate more resources to study bladder cancer. Similarly, the increase in GDP is associated with an increase in research output. We also checked the ratio of population size to the number of publications to better characterize its effect. For example, China, which has the largest population and whose number of publications has massively grown in recent years, still lags behind with one of the lower ratios, unlike Denmark who still produces a significant number of publications. The findings and recommendations of this study are summarized in Table 1.

**Table 1.** Findings and recommendations concerning the current state of bladder cancer research.

Finding	Recommendation
Scarcity of studies focusing on palliative care and screening for bladder cancer	Investing more time in researching these topics because of their important clinical value while allocating more funds for their needs.
The presence of several countries with low number of citations that does not match their significant research output.	Focusing on improving the quality over the quantity of research through careful planning of the projects and increase in funded projects.
Despite the recent significant increase in research funds, funding for bladder cancer remains low.	Increase collaborations of countries that lack supple resources with high income countries to overcome the hurdles and restrictions in research capabilities.
Despite the Covid-19 pandemic research output for bladder cancer did not decrease.	
There is a significant increase in research output noted from US and China. However, when we correlate this to the population size, China has a very low ratio.	

For a comprehensive evaluation of our study, it is important to shed light on its few limitations. As this is a bibliometric study, we relied on the data provided by the used databases to work on our analysis. In addition, our search for publications could have potentially missed a few studies that were not included in our analysis.

## Conclusion

The growing global burden of bladder cancer requires exploration of the current status of bladder cancer research. Facing this growing challenge requires policies that should be enforced through the evidence suggested by research. In this paper, we highlighted the trends and gaps while also attempting to identify factors related to bladder cancer research output around the world.
